# Supplementary material for: Identification and Characterization of Endophytic Fungus DJE2023 Isolated from Banana (Musa sp. cv. Dajiao) with Potential for Biocontrol of Banana Fusarium Wilt
Source: J Fungi (Basel). 2024 Dec 17;10(12):877. doi: 10.3390/jof10120877 (PMC11677757; doi:10.3390/jof10120877)
Supplement: Supplementary file 1 [file jof-10-00877-s001.zip › jof-3293246-supplementary.pdf]

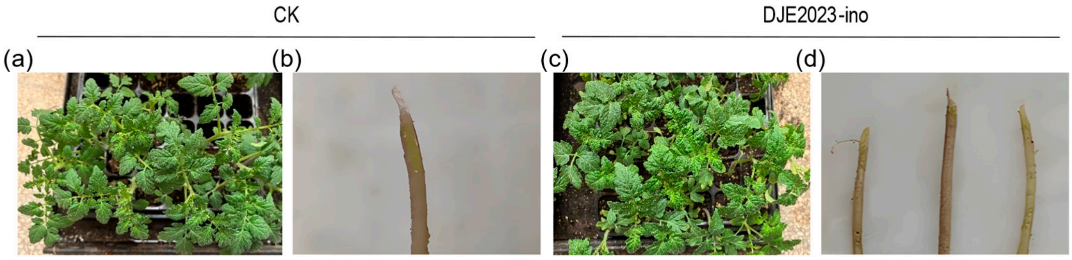

Figure S1. Pathogenicity test of banana endophytic strain DJE2023 on tomato seedings. (a)(c) Leaf. (b)(d) Vascular bundles. CK is water control.

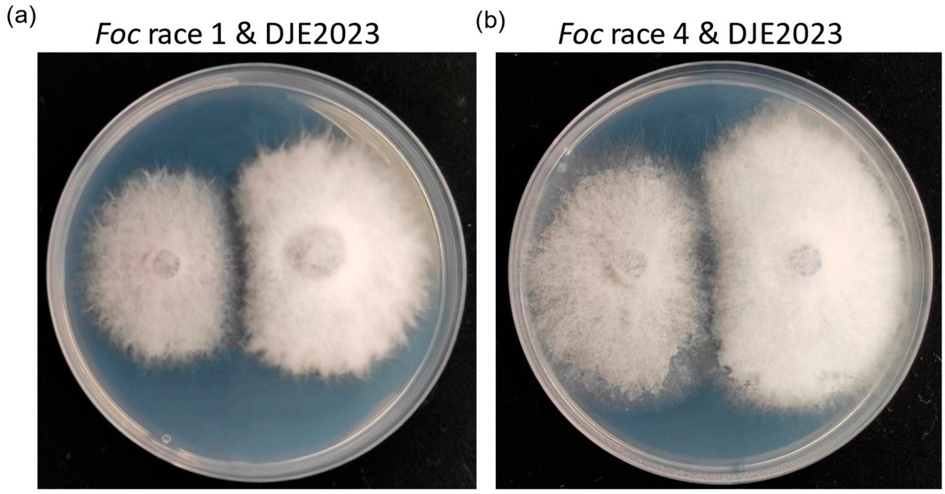

Figure S2. Effect of DJE2023 on *Fusarium oxysporum* f. sp. *cubense* (*Foc*) race 1 and 4. (a) Left: *Fusarium oxysporum* f. sp. *cubense* (*Foc*) race 1, right: DJE2023. (b) Left: *Fusarium oxysporum* f. sp. *cubense* (*Foc*) race 4, right: DJE2023.

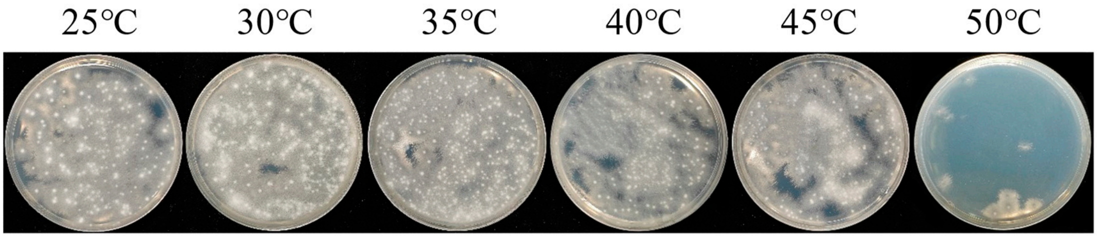

Figure S3. Heat resistance test of DJE2023 conidia.
